# Histological, cellular and behavioral assessments of stroke outcomes after photothrombosis-induced ischemia in adult mice

**DOI:** 10.1186/1471-2202-15-58

**Published:** 2014-05-02

**Authors:** Hailong Li, Nannan Zhang, Hsin-Yun Lin, Yang Yu, Quan-Yu Cai, Lixin Ma, Shinghua Ding

**Affiliations:** 1 Dalton Cardiovascular Research Center, University of Missouri-Columbia, 134 Research Park Drive, Columbia, MO 65211, USA; 2 Department of Bioengineering, University of Missouri-Columbia, Columbia, MO 65211, USA; 3 Department of Radiology, University of Missouri-Columbia, Columbia, MO 65211, USA; 4 Harry S. Truman VA Memorial Hospital, University of Missouri-Columbia, Columbia, MO 65211, USA

**Keywords:** Penumbra, Reactive astrocytes, Brdu labeling, MRI, Behavioral deficits

## Abstract

**Background:**

Following the onset of focal ischemic stroke, the brain experiences a series of alterations including infarct evolvement, cellular proliferation in the penumbra, and behavioral deficits. However, systematic study on the temporal and spatial dependence of these alterations has not been provided.

**Results:**

Using multiple approaches, we assessed stroke outcomes by measuring brain injury, dynamic cellular and glial proliferation, and functional deficits at different times up to two weeks after photothrombosis (PT)-induced ischemic stroke in adult mice. Results from magnetic resonance imaging (MRI) and Nissl staining showed a maximal infarction, and brain edema and swelling 1–3 days after PT. The rate of Bromodeoxyuridine (Brdu)-labeled proliferating cell generation is spatiotemporal dependent in the penumbra, with the highest rate in post ischemic days 3–4, and higher rate of proliferation in the region immediate to the ischemic core than in the distant region. Similar time-dependent generation of proliferating GFAP+ astrocytes and Iba1+ microglia/macrophage were observed in the penumbra. Using behavioral tests, we showed that PT resulted in the largest functional deficits during post ischemic days 2–4.

**Conclusion:**

Our study demonstrated that first a few days is a critical period that causes brain expansion, cellular proliferation and behavioral deficits in photothrombosis-induced ischemic model, and proliferating astrocytes only have a small contribution to the pools of proliferating cells and reactive astrocytes.

## Background

Focal ischemic stroke begins with the blockage of cerebral blood vessels in a certain region of the brain, which can cause brain damage, and human disability and death [1]. In addition to middle cerebral occlusion (MCAo), photothrombosis (PT)-induced ischemia has been widely used as an experimental model to study brain protective mechanism, and *in vivo* cellular responses [2-9]. This model generates infarction with small variability and allows the size and location of ischemic region to be precisely controlled [2-4,10-12].

Besides cell death in ischemic core, ischemia induces a series of alterations in the penumbra at molecular and cellular levels, including cellular proliferation and morphology and gene regulation, in the ipsilateral as well as contralateral hemispheres, especially in the penumbra [12-14]. These alterations are temporal and spatial dependent. The hallmark of molecular and cellular events after ischemia is astrogliosis in the penumbra involved with reactive astrocytes with enhanced expression of glial fibrillary acidic protein (GFAP) and other genes and dramatic morphological changes [12,14]. After a prolonged time following focal ischemia, reactive astrocytes eventually form a glial scar tissue surrounding the infarction [12,15-17]. Microglia are also activated from their resting state after the onset of ischemia. A resting microglial cell has a small cell body with highly branched processes; however, an activated microglial cell exhibits either an enlarged cell body with shorter and fewer processes or elongated processes depending on their distance to ischemic core region [2,18]. Although the responses of astrocytes and microglia to ischemic stroke have been well documented in focal ischemic models, including PT and MCAo models, these studies only analyzed the responses either at certain time points post stroke or in a certain area close to the lesion, or using a qualitative description of histocytocehmistry [12,16,19-23]. Importantly, dynamics of brain damage, cellular proliferation and functional deficits and recovery over time after ischemia was not investigated.

This paper presents a detailed evaluation of stroke outcomes after PT. Using histochemistry, high resolution magnetic resonance imaging (MRI), ‘time-block’ (Bromodeoxyuridine) Brdu labeling, immunostaining and behavioral tests, we assessed stroke outcomes over a period of two weeks after PT. The progress of ischemic damage including infarct volume, brain edema and swelling was assessed using histochemistry and MRI. Spatial and temporal changes in cellular proliferation and glial scar formation were studied using immunostaining. Behavioral tests were performed to assess the functional deficits and recovery after PT. Our results show that the rate of generation of proliferating Brdu+ cells is time and regional dependent, and proliferating reactive astrocytes account for a small percentage of all Brdu+ reactive astrocytes. Importantly, we found cell proliferation and reactive astrocyte and microglia generation occur at the highest rate within post ischemic days 3 to 4, which is concomitant with the time period of infarction expansion and the highest behavioral deficits. Our study suggests that first a few days is a critical period that causes brain expansion, cellular proliferation and behavioral deficits in this mouse model of stroke and provides insights for interventions to improve stroke outcomes in experimental and clinic studies of stroke therapy.

## Methods

### Animals

Adult male C57BL/6 J mice aged 2 months were used in this study. All procedures were performed according to the NIH Guide for the Care and Use of Laboratory Animals and were approved by the University of Missouri Animal Care Quality Assurance Committee.

### Photothrombosis (PT)-induced brain ischemia model

PT was performed similarly as previously described [2,12]. Briefly, mice were anesthetized by ketamine and xylazine (130 mg/10 mg/kg body weight), and rose Bengal was injected through the tail vein with a dose of 30 mg/kg body weight. An area of 1.5 mm in diameter in the somatosensory cortex was focally illuminated on the intact skull for 2 min through a 10× objective by a green light (540–580 nm) from an X-cite 120 PC metal halide lamp (EXFO, Canada) with a power of 12%. To maintain body temperature at 37°C during the procedure, mice were placed on a warm heating pad (Fine Science Tools, CA).

### Titration of proliferating cells using 5-bromo-2-deoxyuridine (Brdu) labeling

To study the time course of cell proliferation at different times after ischemia, Brdu (B9285, Sigma) was administered in seven groups of mice (5 per group) through intraperitoneal (IP) injection once daily with a dose of 50 mg/kg at the beginning of days 1, 3, 4, 5, 9, 11, and 13 after PT for two consecutive days [12]. For control mice, Brdu+ was injected once daily for two days. Mice were transcardially perfused one day following the last injection. This protocol allowed us to determining the cumulative number of proliferating cells within a two-day period and the relative rate of proliferating cell generation after different times from the onset of PT. We termed this method ‘time-block’ Brdu labeling protocol.

### Transcardial perfusion, infarct volume measurement, immunostaining and cell counting

Mice were transcardially perfused using 4% paraformaldehyde (PFA) after PT with similar procedures described in our previous studies [2,11,12]. Coronal sections of 30 μm were cut using a cryostat (CM1900, Leica). For infarct volume measurement, brain sections were stained by 0.25% cresyl violet (Nissl). Images were acquired using a 4× objective under a bright-field microscope. The areas of infarction were delineated and quantified using ImageJ software (NIH) and infarct volume was calculated by summation of the lesion areas of all slices and integrated by the slice thickness [2,11,12].

For the immunostaining of brain section for Brdu, GFAP, Iba1 and NeuN, the procedure was described in our previous studies [2,11,12]. Rat anti-Brdu antibody (1:200 ~ 400, Abcam), rabbit polyclonal anti-GFAP (1:300, Millipore), rabbit anti-Iba1 (1:500, Wako), and rabbit anti-NeuN (1:300, Millipore) primary antibodies were used. FITC- or rhodamine-conjugated anti-rabbit IgG were used as secondary antibodies. Fluorescent images were acquired using a Nikon epi-fluorescence microscope (Nikon, NY) or an Olympus FV1000 confocal fluorescent microscope.

The numbers of Brdu+, GFAP+, Iba1+, and NeuN+ cells in the penumbra were counted using the MetaMorph imaging software as in our previous studies [2,12]. For contralateral side of ischemic mice, the number of cells was counted in the mirror region of the same size. For non-ischemic mice, number of cells was counted in the equivalent region of the same size. The data from 3–4 slices of each mouse brain were averaged as one value and values from five mice in each group were averaged as a group value.

### Magnetic resonance imaging (MRI) and analysis

A group of 4 mice were imaged at 5 h, 1, 3, 6, 10 and 14 days after PT. MRI experiments were carried out on a horizontal-bore 7 Tesla Bruker AVANCE III BioSpec MRI system using a 35 mm inner diameter birdcage quadrature radio frequency coil (Bruker Corp., MA). Mice were anesthetized using isofluorane (1.5% in oxygen) and maintained in anesthetized state during the experiments. The changes in respiratory rate were monitored using a small animal monitoring and gating system (SAII, NY) in order to adjust the anesthetic concentration. T_2_-weighted images (T_2_WIs) were acquired using image parameters echo time/repetition time (TE/TR) = 40/2300 ms. Diffusion-weighted images (DWIs) were acquired with TE/TR = 35/4000 ms using a B value of 800 s/mm^2^ in slice direction. Apparent diffusion coefficients (ADCs) in mm^2^/s were calculated by pixel-by-pixel from DWIs [24]. For both sequences, the field of view was 30 × 30 mm^2^ for 16 slices, each with a thickness of 0.8 mm. The matrix size was 256 × 256 for T_2_WIs and 128 × 128 for DWIs.

Quantitative MRI analysis was performed using ImageJ software. Brain swelling was assessed by the ratio of the ipsilateral (IH) volume to contralateral hemisphere (CH) volume from T_2_WIs, i.e., IH/CH [25]. The ischemic lesions with hyperintense signal in T_2_WIs and DWIs were manually delineated by a blinded observer on each slice for lesion area calculation. Infarct volumes were calculated by summation of the lesion areas of all slices and integrated by slice thickness (0.8 mm) with correction of brain swelling by multiplying IH/CH [24-26]. The ratio of ADC value of the ischemic region to ADC value of the mirror region in the contralateral hemisphere was calculated to characterize tissue damage in term of vasogenic and cytotoxic edema [24].

### Behavior assessment

Behavioral tests were performed on control (N = 8) and ischemic mice (N = 10) on day 2, 4, 6, 10 and 14 after PT. Pre-training was done before ischemia. These tests included:

a) *The cylinder test*. The mouse was placed in a transparent cylinder 10 cm in diameter and 15 cm high. Use of Forelimb use to touch the wall was recorded. A total of 20 movements were recorded for each test. The final score = (non-impaired forelimb movement - impaired forelimb movement)/(non-impaired forelimb movement + impaired forelimb movement + movement with both limbs) as previous described.

b) *The hanging wire test*. Mice utilized forelimbs to hang their body weight on a wire stretched between two posts 60 cm high. The time until the mouse dropped off the wire was recorded. A time of zero was assigned if the mouse dropped off immediately while 120 s was the maximum period. Two trials were performed for each mouse at each time point.

c) *The pole test*. Adhesive tape was placed on the surface of the pole to avoid sliding. A mouse was placed on the top of a 60 cm vertical pole with a diameter of 10 mm. Time taken to reach the ground was recorded. If the mouse stopped during a run, then the trial was excluded and repeated.

d) *The adhesive removal test.* A small ‘adhesive patch (rectangular 0.35 × 0.45 cm)’ was applied to the forelimb contralateral to the photothrombotic hemisphere. The mouse was then put into a cage. Time taken to contact and remove adhesive tape was recorded with 120 s being the time limit. Contact occurred when the mouth was first used to remove the patch.

### Statistical analysis

Quantitative data were expressed as mean ± s.e.m. Statistical comparisons were made by a student’s paired *t*-test for two groups or a one-way ANOVA (Bonferroni post hoc test) for multiple groups. *p* < 0.05 was considered statistically significant.

## Results

### Evolvement of ischemic lesion after ischemia

#### Ischemic infarction evaluated by Nissl staining

**Figure 1:**
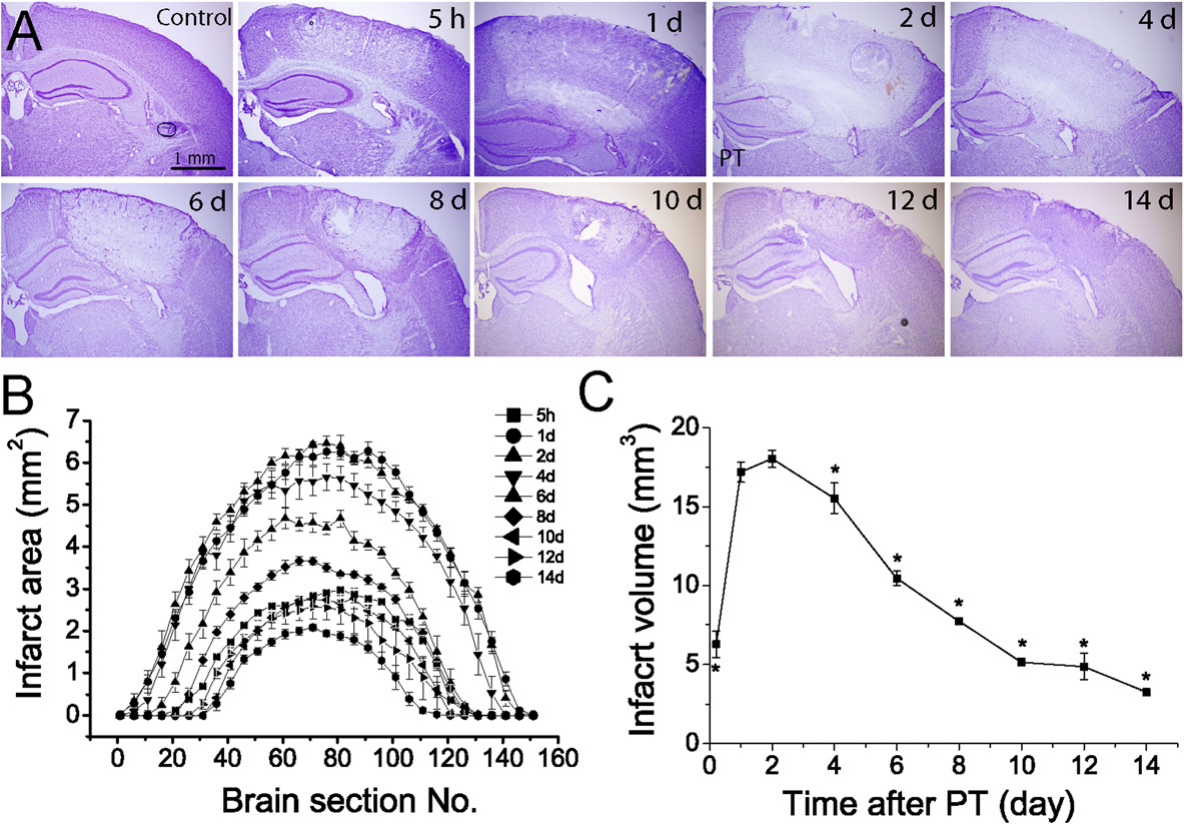
**Infarction development in mouse brains after PT. A)** Representative Nissl-stained brain sections showing the lesion in the middle of infarction at different times after PT. **B)** Infarct areas of rostrocaudal series of brain sections at different times after PT. **C)** Time course of infarct volumes after PT. Data were averaged from values of five mice from each group. N=5 mice in each time point. *p<0.05, compared with value at day 2 after ischemia, ANOVA test.

To study the evolvement of ischemic lesion, nine groups of mice (N = 5 for each group) were sacrificed 5 h, 1, 2, 4, 6, 8, 10, 12 and 14 days after PT. Nissl staining showed that the lesions were discernible 5 h after PT and clearly demarcated from the surrounding region of ischemic core one day after PT (Figure 1A). The underlying white matter was also partly affected. A newly formed barrier of glial scar separating the normal brain tissue from the ischemic core was developed on the sixth day after PT. Infarct volume increased from 5 h and reached a maximum one to two days after ischemia and then gradually reduced (Figure 1B-C) suggesting delayed infarction expansion and spontaneous recovery from ischemic brain injury.

### Ischemic damage evaluated by serial MRI

**Figure 2:**
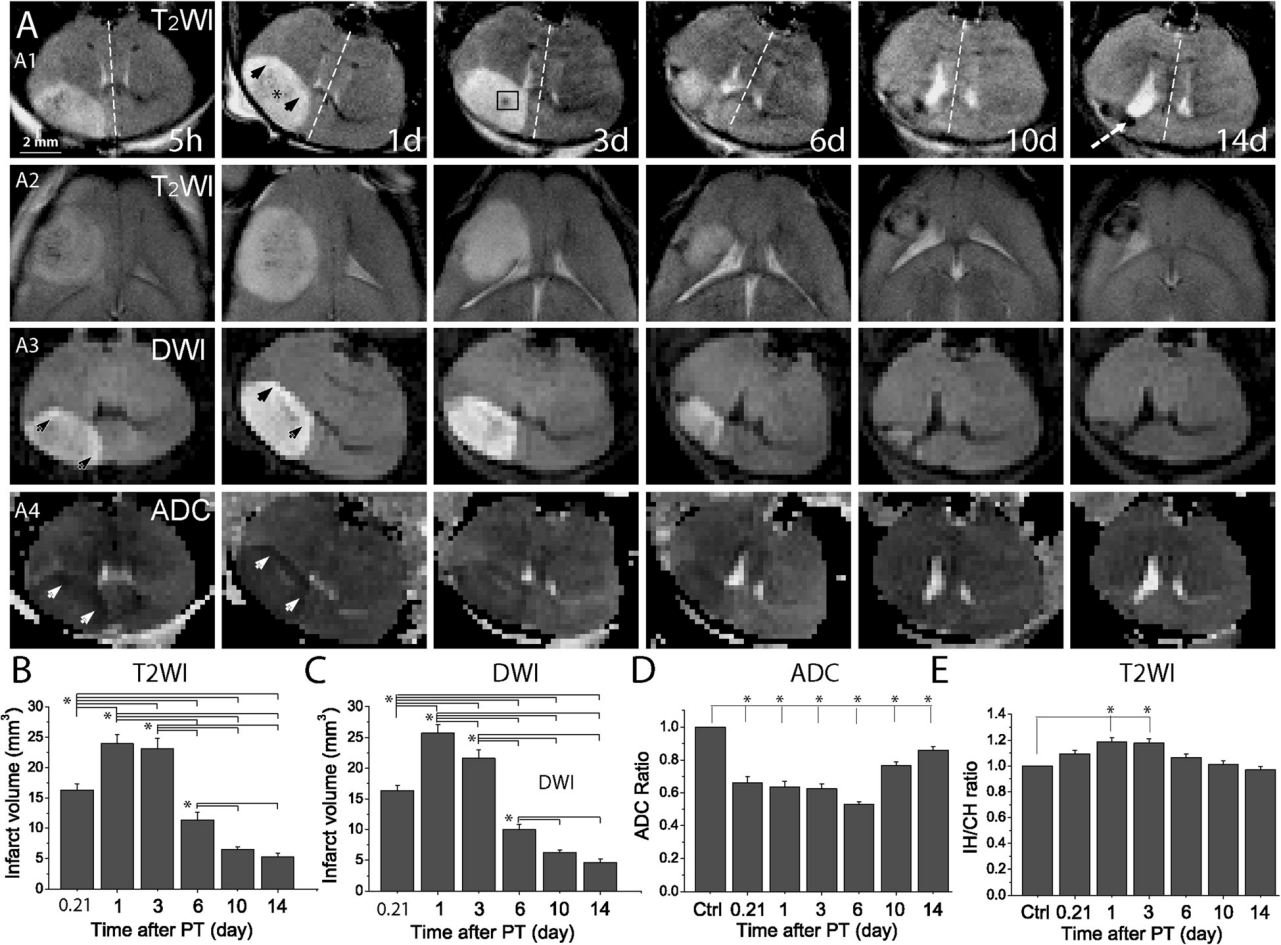
**T**_**2**_**-weighted images (T**_**2**_**WIs), diffusion-weighted images (DWIs), and ADC maps after PT and analysis of brain damage. (A)** Serial T_2_WIs, DWIs and ADC maps of a representative mouse at 5 h, 1, 3, 6, 10, and 14 days after ischemia. In T_2_WIs (A1), arrowheads indicate the hyperintense signal in penumbra, star isointense signal in ischemic core, and dashed line arrow indicates glial scar formation. The boxed region in T_2_WI on day 3 was a hemorrhagic transformation. The midlines are indicated by dashed lines in T_2_WIs. In DWIs and ADC maps (A3-A4), arrowheads indicate vasogenic and cytotoxic edema. **(B-C)** Summary of brain infarct volumes based on T_2_WIs and DWIs. **(D)** The brain edema progression calculated as the ratio of ADC values in the ischemic region to the ADC values in the mirror region in contralateral hemisphere. **(E)** Time course of the ratio of ipsilateral to contralateral hemisphere volumes (IH/CH) based on T_2_WIs. All the data were averaged values from 4 mice. *p<0.05, ANOVA test. In **B-C**, comparisons are indicated in the figures, and in **D-E**, values were compared with control.

One of the advantages of MRI is that the progressive changes of ischemic damage can be measured *in vivo* over time in the same live mice. T_2_WIs, DWIs and ADC maps were used to detect the ischemic brain injury. Consistent with histological study, the lesion was evident 5 h after ischemia, featured by heterogeneous hyperintense signals on T_2_WIs and DWIs (Figure 2A). Infarct volumes were analyzed based on T_2_WIs and DWIs (Figure 2B-C). An increase in infarct volume was found from 5 h to 1 day and persisted through day 3 post ischemia, after which the lesion then gradually decreased (Figure 2B-C). There were two different signal regions identified in the T_2_WIs: 1) the hyperintense signal in the periphery of the lesion (arrowheads in Figure 2A:A1) and 2) the isointense signal in the core of the lesion (the star in Figure 2A:A1). These two regions are equivalent to the penumbra and ischemic core revealed by Nissl staining. Strong focal hypointense signals (boxed region) often appeared within ischemic core on T_2_WI after day 3 following PT, and were identified by Nissl staining as small hemorrhagic transformations [25]. Strong decline of hyperintense signals was observed on T_2_WIs and DWIs after day 6. Up until day 10 after PT, a strong hypointense signal in T_2_WI (dashed line arrow) and a corresponding area in DWI were observed and identified as the location of the surrounding glial scar tissue by Nissl staining. The vasogenic and cytotoxic edema was identified by enhanced hyperintense signals in DWIs and decreased hypointense signals on ADC maps, especially at acute phase of stroke, i.e., 5 h to 3 days after PT (arrowheads, Figure 2A:A3-4). Brain edema, analyzed by the intensity ratio of ipsilateral side to contralateral side based on ADC maps, was significantly developed in a very acute phase 5 h after PT, and a sub-acute phase 1, 3, and 6 days after PT (Figure 2D). The decrease of ADC ratios from 5 h to 6 days after PT indicates a delayed brain edema formation after the stroke onset. ADC values in the chronic phase of day 10 to 14 post ischemia were increased compared to the value on day 6 although they were still significantly lower than the control. This may reflect the contribution of reduced edema and glial scar tissue formation. Similarly, based on the volumes of IH and CH from T_2_WIs, significant cerebral swelling was observed at 5 h and the swelling further increased on day 1 and remained at a high level on day 3 after PT (Figure 2E). After day 6 post PT, the volume of ipsilateral hemisphere was slightly smaller than contralateral hemisphere, suggesting the occurrence of tissue shrinkage or loss and brain atrophy in the chronic phase of ischemia. These results were consistent with the observation that the midline of the brain was significantly shifted over to the side of contralateral hemisphere from 5 h to day 3 post PT before shifting back to the middle of brain after day 6 following PT (Figure 2A:A1). Thus, multiple parameters, including brain infarct volume, brain edema and swelling, were obtained from MRI study, demonstrating that MRI is a valuable tool to study lesion progression and brain damage in this mouse model of ischemic stroke. Results from both histological and MRI studies showed infarction increases from very acute phase at 5 h and remains persistent in sub-acute phase from days 1 to 3 post ischemia.

#### Titration of cell proliferating rate at different times after PT

**Figure 3:**
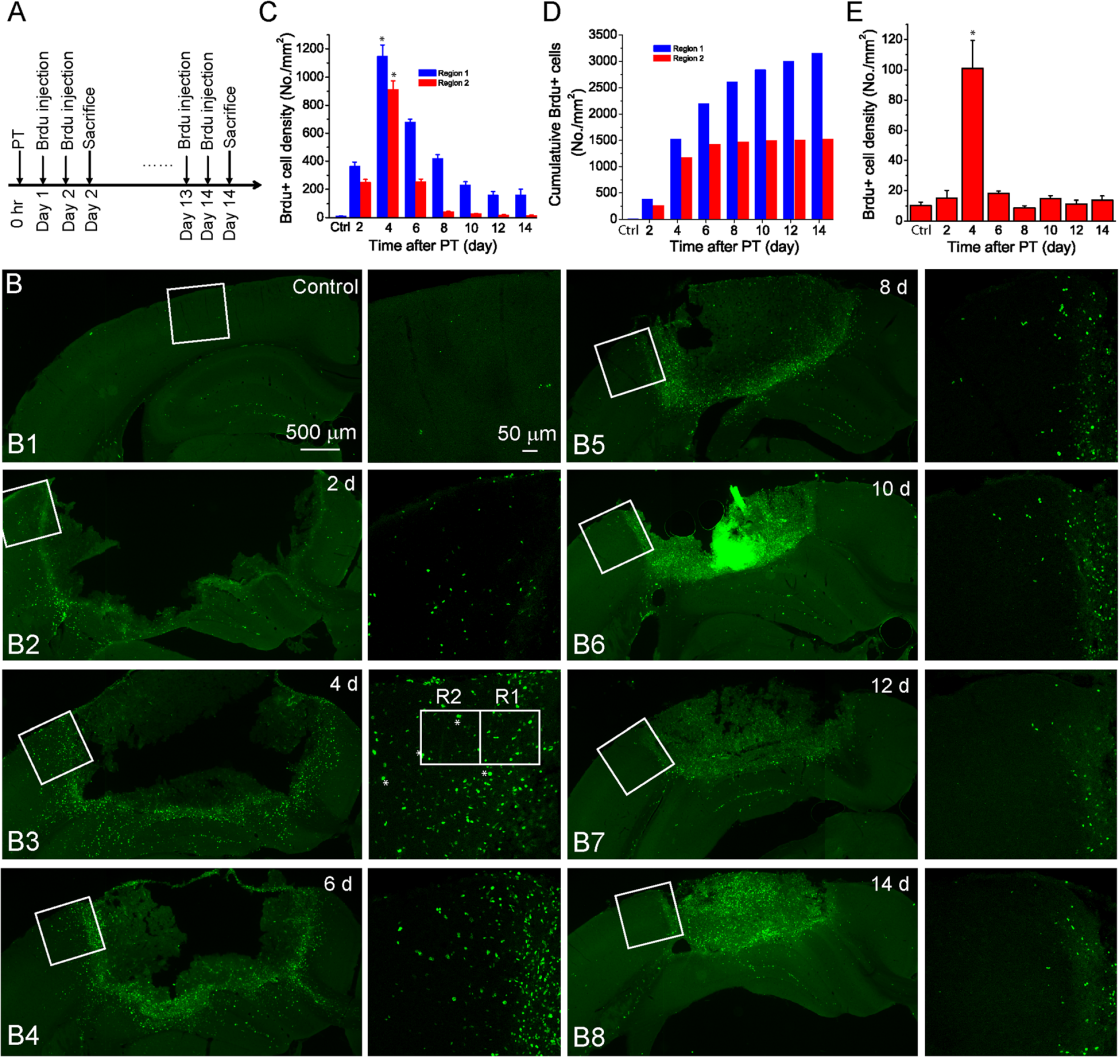
**Titration of proliferating cells after PT. A)** ‘Time-block’ Brdu injection protocol to titrate cell proliferation rate after PT. Brdu were administered through intraperitoneal (IP) injection in seven groups of mice (N=5 mice in each group) at beginning of days 1, 3, 5, 7, 9, 11, and 13 after PT for 2 consecutive days, and mice from each group were sacrificed one day after last injection. **B)** Representative Brdu-stained brain sections from mice at different times after PT. **C)** Summary of Brdu+ cell density presented as cell number per mm^2^ in the two regions with an area of 200 μm × 200 μm in the layers 2/3 of cortex. Region 1(R1) is located 0-200 μm from the edge of ischemic core, and Region 2 (R2) is located 200-400 μm from the edge of ischemic core (see the left panel of B3). **D)** Cumulative Brdu+ cell density. **E)** Summary of Brdu+ cell density in the contralateral side in the equivalent location of ischemic core. Brdu+ cells are uniformly distributed in the cortex. N=5 mice in each time point. *p<0.05, value on day 4 compared with other dates after ischemia and control using ANOVA test.

Focal ischemic stroke induces cell proliferation and glial scar formation. To determine the dynamics proliferating cell and the rate of cell proliferation after stroke, we designed a ‘time-block’ Brdu injection protocol to titrate proliferating cells in the penumbra. In this protocol, Brdu was injected in seven groups of mice (N = 5) at the beginning of days 1, 3, 5, 7, 9, 11 and 13 after PT for consecutive 2 days (Figure 3A). Mice were sacrificed one day following the last injection. To quantify proliferating cells, Brdu+ cells were counted from fluorescent images in two different regions with an area of 200 × 200 μm in the penumbra of layers 2/3 of the cortex. One region (R1) was immediate to ischemic core and the other region (R2) was 200 μm from ischemic core (see Figure 3B:B3). The number of Brdu+ cells represents the cumulative proliferating cells within two days from Brdu+ injection to transcardial perfusion. Therefore, they also represent the relative rate of proliferating cells generated within a 2-day time interval at different times after PT. The densities of Brdu+ cells in R1 & R2 were largely increased from post ischemic day 1 to 2 after PT as compared with the control and were further dramatically increased from day 3 to 4 after PT, when the highest levels were reached (Figure 3C). The densities of Brdu+ cells then decreased over time in both regions (Figure 3C-D). Notably, the density of Brdu+ cells in R1 was higher than the density in R2 in each time interval post ischemia. Interestingly, a low number of Brdu+ cells was observed in the contralateral hemisphere, with the highest number produced from post ischemic day 3 to 4 after PT, but there was no significant difference in other time periods (Figure 3E, Additional file 1: Figure S2). These results demonstrate that the rate of proliferating cell generation in the penumbra after ischemia is highly spatiotemporal dependent.

#### Dynamic activation and proliferation of astrocytes, and glial scar formation after PT

**Figure 4:**
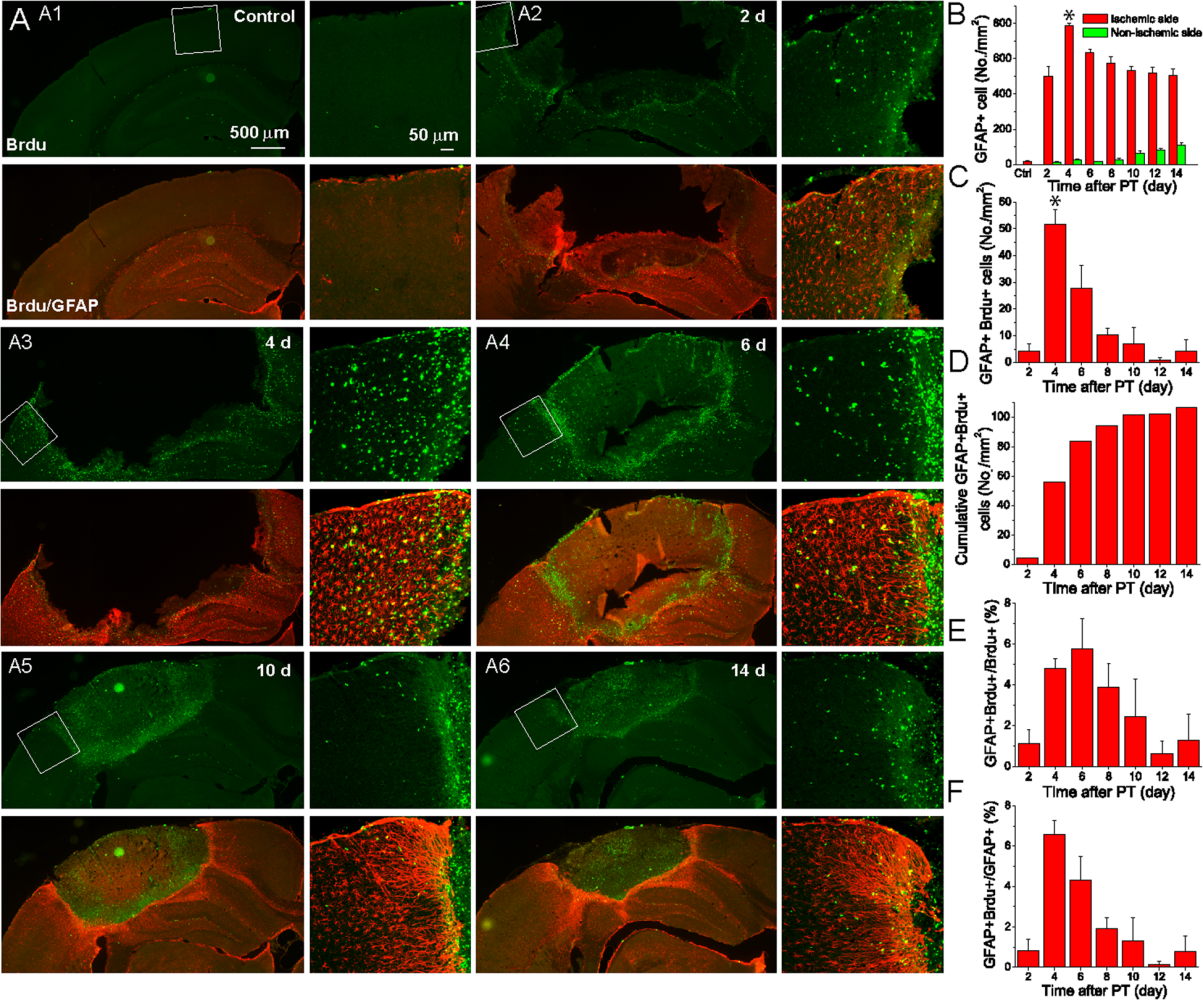
**Dynamic activation and proliferation of GFAP+ astrocytes after PT. A)** Representative fluorescent images of GFAP and Brdu double stained brain sections from mice at different times after PT. Right panels are the high resolution confocal images of boxed regions in the left panels. **B)** The density of GFAP+ cells in the penumbra. **C-D)** The density and cumulative density of GFAP+Brdu+ cells in the penumbra. **E)** The percentage of GFAP+Brdu+ double stained cells out of total Brdu+ cells in the penumbra. **F)** The percentage of GFAP+Brdu+ double stained cells out of total GFAP+ cells in the penumbra. In Figure **(E-F)**, the cells were counted in the penumbral region of 200 μm × 400 μm in layers 2/3 cortex located 0-400 μm from the edge of ischemic core. N=5 mice in each time point. *p<0.05, compared with value at day 4 after ischemia using ANOVA test.

Astrogliosis is a hallmark of damaged brain tissue after a stroke and is characterized as progressive changes in proliferation, gene expression and cellular morphology [13,14,16,19,20]. To study the dynamic change of reactive astrocyte generation and the contribution of reactive astrocytes to proliferating cells, we used double staining of GFAP and Brdu to evaluate the percentage of proliferating astrocytes out of total proliferating cells, i.e., Brdu+ GFAP+ cells/Brdu+ cells. Under normal conditions, astrocytes in mouse cortex usually express little GFAP [2,12] (Figure 4A:A1). We first counted GFAP+ cells in a region of 200 × 400 μm in layer 2/3 in the penumbra. Two days after PT, GFAP was significantly upregulated, and much more GFAP+ astrocytes emerged as compared with the control mice (Figure 4A-B). The GFAP+ cell density reached a peak value on day 4 after PT (786 ± 15 cell/mm^2^) and was maintained at a high level until day 14 after PT, the longest time point in this study (Figure 4A-B). Small increases of GFAP+ astrocytes in the contralateral hemisphere at each time point after PT were also observed (Figure 4B). GFAP and Brdu double stained (GFAP+ Brdu+) astrocytes were initially undetectable in control mice, but their numbers reached a peak of 52/mm^2^ from post ischemic days 3 to 4 after PT and then decreased over time (Figure 4C-D), suggesting that proliferating GFAP+ astrocytes are generated with the highest rate in this time period after PT. Nevertheless, the GFAP+ Brdu+ astrocytes only accounted for a small percentage out of total Brdu+ cells in each time interval with a significant increase of 4.8 ± 0.5% within post ischemic days 3 to 4 after PT, reaching the highest level of 5.8 ± 1.5% within days 5 to 6 before gradually decreasing thereafter (Figure 4E). On the other hand, the ratio of GFAP+ Brdu+ to GFAP+ reached 6.6 ± 1.6% within days 3 to 4 after PT (Figure 4F). These results demonstrated that stroke induces an increasing number of proliferating cells and reactive astrocytes following a time-dependent manner. The fact that there was only a small percentage of GFAP+ Brdu+ cells from total Brdu+ cells indicates that proliferating reactive astrocytes only account for a small portion of the total proliferating cells. On the other hand, the small percentage of GFAP+ Brdu+ cells from total GFAP+ cells indicates that astrogliosis mostly is due to the upregulation of GFAP from existing astrocytes. Nevertheless, this Brdu labeling protocol underestimated the total number Brdu+ cells since a single daily injection will not label all proliferating astrocytes that might divide during a one-day period [27].

Morphologically, GFAP+ astrocytes in the penumbra experienced significant changes over time after ischemia. Up to day 4 post PT, astrocytes exhibited a stellate morphology even though GFAP expression was highly upregulated (Figure 4A:A2-A3). However, starting from day 6, astrocytes in the penumbra were densely packed and formed a stream with their elongated (straight) processes pointing towards the ischemic core, i.e., astroglial scar tissue was formed (Figure 4A:A4). After day 10, the morphology of astrocytes at the scar border was similar, suggesting the maturation of astroglial scar tissue (Figure 4A:A5-A6); however, the morphology of astrocytes in the distant regions from the penumbra were similar to the astrocytes in the control condition. Thus, reactive astrocytes exhibited heterogeneity in morphology and GFAP expression levels, and such heterogeneity was spatiotemporal dependent. The ischemic core, the central region surrounded by reactive astrocytes and glial scar tissue, on the other hand, contained no GFAP+ astrocytes after day 2 suggesting the demise of astrocytes.

#### Dynamic activation and proliferation of Iba1+ cells after PT

**Figure 5:**
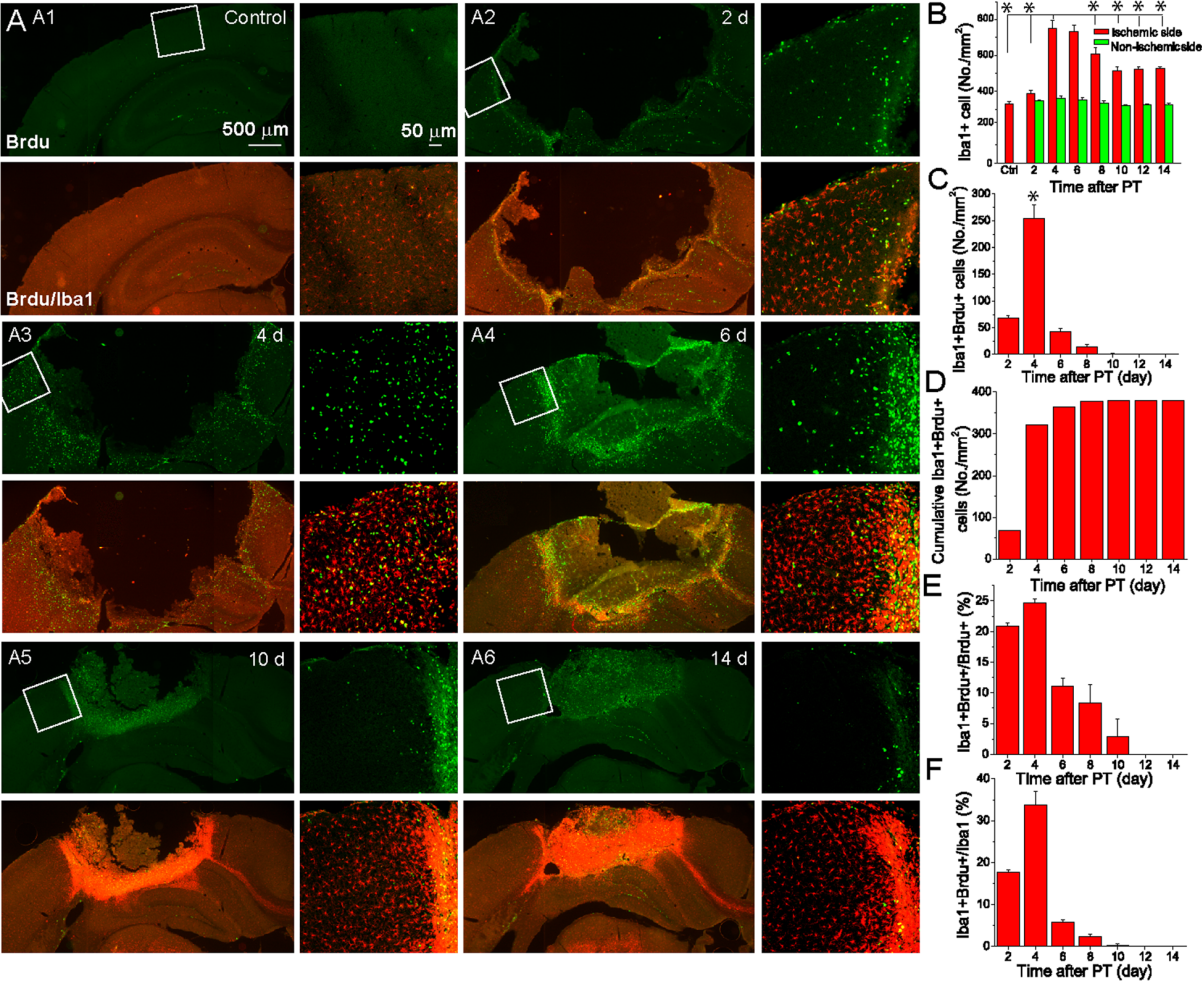
**Dynamic activation and proliferation of Iba1+ cells after PT. A)** Representative fluorescent images of Iba1 and Brdu double stained brain sections from mice at different times after PT. Right panels are the high resolution confocal images of boxed regions in the left panels. **B)** The density of Iba1+ cells in the penumbra. **C-D)** The density and cumulative density of Iba1+Brdu+ cells in the penumbra. **E)** The percentage of Iba1+Brdu+ double stained cells out of total Brdu+ cells in the penumbra. **F)** The percentage of Iba1+Brdu+ double stained cells out of total Iba1+ cells in the penumbra. In Figure **(E-F)**, the cells were counted as in Figure 4. N=5 mice in each time point. *p<0.05, compared with value at day 4 after ischemia using ANOVA test.

Iba1 is a marker for resting and activated microglia/macrophages [14]. To investigate the dynamic responses of microglia/macrophages after PT, we performed Iba1 and Brdu double staining. Activated Iba1+ and amoeboid Iba1+ cells were observed two days after PT in the penumbra and ischemic core (Figure 5A:A2). There was a significant increase in Iba1+ cells in the penumbra from post ischemic day 2 to day 4 (Figure 5A-B). The density of Iba1+ cells reached the highest level 4 days after PT, and maintained at a high level after day 8. The densities of Iba1+ cells from non-ischemic side remained consistent over time after PT. Double immunostaining showed that there was the highest density of Iba1+ Brdu+ cells in the penumbra within post ischemic days 3 to 4 (109 ± 2 cells/mm^2^), and the density decreased sharply from day 5 after PT (Figure 5C-D). Iba1+ Brdu+ cells were undetectable after day 10. The percentage of Iba1+ Brdu+ double positive cells out of the total Brdu+ cells had a highest value of 24.6% within post ischemic days 3 to 4 (Figure 5E) and reduced sharply thereafter. Iba1+ Brdu+ cells were undetectable after post ischemic day 12. On the other hand, the ratio of Iba1+ Brdu+ double stained cells to Iba1+ cells reached 33.8 ± 7.1% within post ischemic days 3 to 4 (Figure 5F). Since infiltrated cells do not proliferate [28], Iba1+ Brdu+ cells are proliferating cells from resident microglia. These results demonstrated that ischemic stroke induces a fast and sharp increase of proliferating Iba1+ microglia in the penumbral region within a narrow time window as compared with generation of GFAP+ Brdu+ cells. Overall, the density of Iba1+ Brdu+ cells is higher than GFAP+ Brdu+ cells before day 4 after PT. Contrary to GFAP+ staining, densely packed Iba1+ cells in the ischemic core region were observed 6 days after PT (Figure 5A:A5-A6). Many of these cells were Brdu+ and belonged to inflammatory, fibrotic and other types of cells [27,29].

#### Neuronal responses after PT

NeuN is located in the nuclei of neurons in the normal brain and is used for detecting the injury of neurons in ischemic brains [30]. After photothrombosis, there was a slight but significant reduction of the density of NeuN+ cells in the penumbra at different times (Additional file 1: Figure S1A-B). However, no colocalized NeuN and Brdu signal was detected at each time point indicating that no new mature neurons were generated after ischemia in the penumbra based on our Brdu+ labeling protocol. This is consistent with a report from Haupt et al. [16]. The results suggest that glial scar functions as a protector to keep the healthy neurons in the distant region to ischemic core from being further damaged at early stage.

#### Behavior assessment

**Figure 6:**
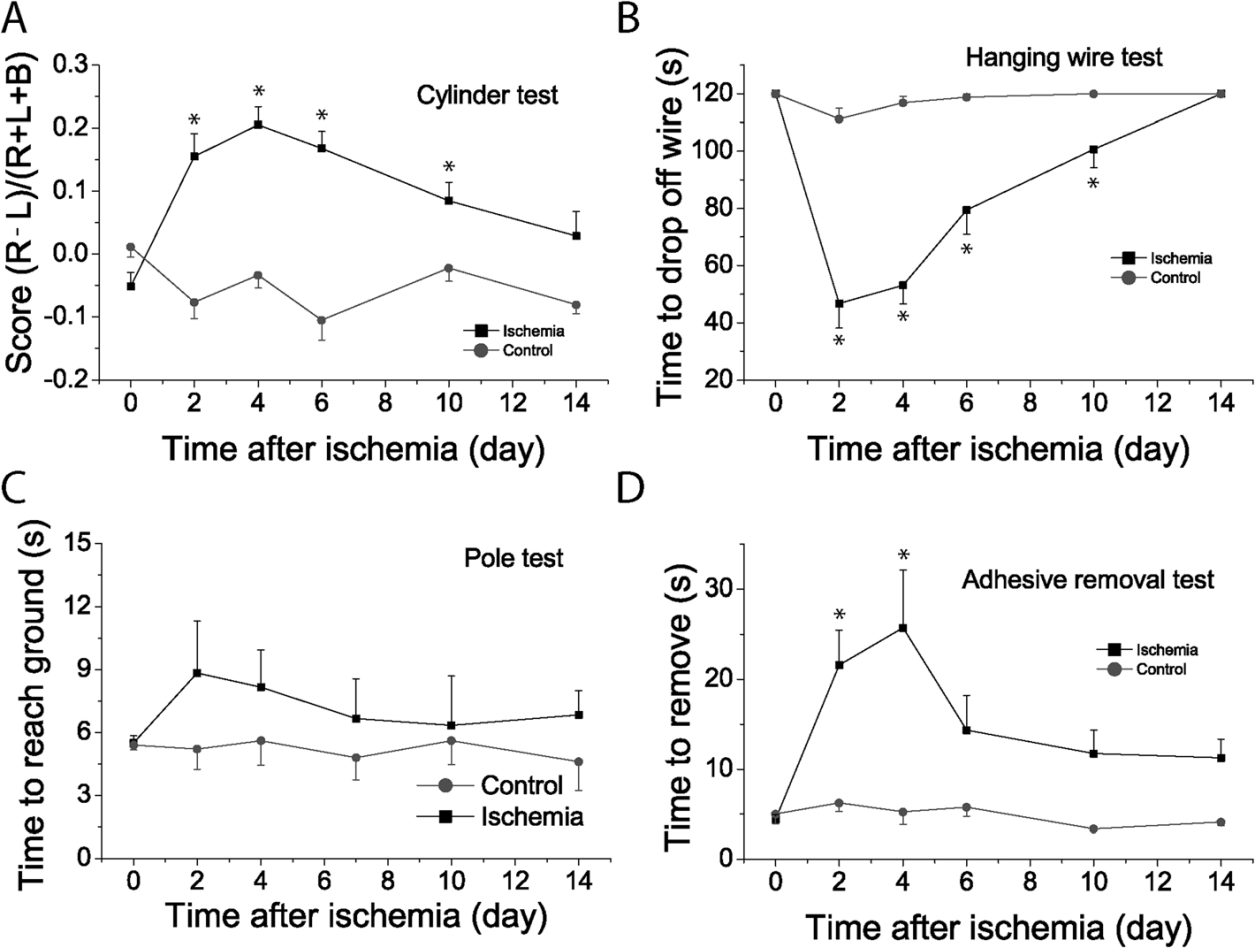
**Behavior assessments of sensory-motor function after PT.** Four behavioral tests, i.e, cylinder **(A)**, hanging wire **(B)**, pole **(C)** and adhesive removal **(D)** tests were performed on sham and ischemic mice to assess functional deficits and recovery over a time course of two weeks after PT. The value of day 0 is the data from pre-ischemic mice for ischemic group in each test. N=8 and 10 for ischemic and control mice. *p<0.05, compared with pre-stroke value within stroke group using ANOVA test.

Although the infarct volume is an essential and precise measurement of stroke outcome, the most meaningful outcome parameter of cerebral ischemia is the functional deficit measures using behavioral tests. To test whether functional deficits after PT are closely correlated with infarction expansion and cellular proliferation, we conducted four behavioral tests after PT on sensory-motor tasks: cylinder, hanging wire, pole, and adhesive removal tests. We conducted behavioral tests prior to PT to establish a basal level, and 2, 4, 6, 10, and 14 days after PT. The cylinder test was conducted to evaluate the asymmetricity of forelimb use for weight shifting during vertical exploration and assesses motor deficits after an ischemic stroke [31]. Data analysis showed that stroke mice used their contralateral paw much less frequently than the control group mice with statistical differences on days 2, 4, 6 and 10 after ischemia (Figure 6A). PT-induced damage on sensory-motor cortex significantly affected asymmetric shifting ability, and the functional deficits could gradually improve after stroke (Figure 6A). To evaluate the grasping ability and forelimb strength after ischemia, the hanging wire test was performed [31]. There was a significant difference between stroke and control mice at days 2, 4, 6 and 10 after ischemia (Figure 6B). The deficit reached high levels from days 2–4 and stroke mice then rapidly recovered from the deficit of grasping ability. The pole test was used to evaluate simple motor function after a stroke [32]. Stroke mice appeared to need more time to reach the ground than control mice, even though there was no significant difference between the two groups (Figure 6C). To further evaluate sensory-motor deficits involving the sensory-motor cortex, cortico-spinal tract, and striatum after ischemia, we also performed the adhesive removal test [33]. Stroke mice needed a much longer time to remove the adhesive dots than control mice on day 2 and 4 after PT (Figure 6D). After day 6, stroke mice had significant functional recovery although they still needed a longer time to remove the adhesive dots than the control group (Figure 6D). Overall, our study on four different behavioral tests demonstrated that the largest functional deficits occurred from days 2 to 4 after ischemia when the largest infarction was developed and the highest rate of cellular proliferation was reached. Significant functional recovery starts after day 6 of post stroke when glial scar starts forming and infarction is decreased. Mice gradually and spontaneously recovered thereafter up to 14 days after stroke. Results from behavioral tests demonstrate that functional deficits are closely correlated with brain damage and cellular proliferation.

## Discussion

Using multiple approaches, the following findings were observed after PT-induced ischemia: 1) Histological and serial MRI data showed that brain infarction can be observed as early as 5 h after PT, and reached a peak on days 1–3 after PT before declining thereafter. Significant brain swelling and edema were observed *in vivo* in the same time frame after PT from MRI. 2) The Brdu labeled proliferating cells exhibit temporal and spatial changes after PT. The rate of Brdu+ cell generation is higher in the region close to ischemic core and reaches the highest level within post ischemic days 3 to 4. Similar time courses were observed for the generations of proliferating GFAP+ astrocytes and Iba1+ microglia/macrophages. 3) The percentage of GFAP+ Brdu+ reactive astrocytes out of total Brdu+ cells was small in each time interval, with the highest rate of ~6% within post ischemic days 5 to 6. 4) The largest sensory-motor functional deficits were observed on post ischemic days 2–4 with beginning recovery detected thereafter. Thus, the current study found that the first a few days is a critical period that causes brain expansion, cellular proliferation and behavioral deficits after PT and functional deficits are positively correlated with brain infarct expansion as well as swelling and edema, and cellular proliferation.

Our studies from histochemistry, and T_2_WIs and DWIs of MRI of live mice indicate that infarction expanded to a maximum 1–3 days after the onset of PT and gradually reduced over the time course of two weeks. Nissl staining showed clear glial scar formation around the border of ischemic region six days after PT. Based on ADC maps and T_2_WIs, we observed significant vasogenic and cytotoxic edema and brain swelling within the first three days after PT, and a significant decrease and atrophy after day 6. Data of a time-dependent decline in lesion and swelling after day 6 from MRI, together with Nissl staining, indicate that the expansion of infarction was accompanied by brain edema development. The spontaneous recovery of brain injury and glial scar formation begum from about day 6, consistent with other reports [21,34]. Our results are consistent with clinical study using serial MRI on patients after stroke, which revealed an increase in stroke volume from several hours to 3–5 days after stroke onset [35] Interestingly, both T_2_WIs and DWIs can detect ischemic lesion as early as 5 h after PT when demarcation is not clear and penumbral ischemic tissue is salvageable using tPA in clinic, suggesting that MRI is a useful tool for the detection of very acute ischemia.

Astrocytes experience a variety of alterations in brain disorders [36]. Astrocytes can exhibit enhanced Ca^2+^ signaling and are known to become reactive over time after ischemia as characterized by an excessive expression of GFAP (i.e., astrogliosis), and eventually form a glial scar surrounding the area of the ischemic core [5,16,29]. Reactive astrocytes could be formed from the transformation of existing astrocytes or newly generated astrocytes with a common feature of high GFAP expression levels [12,16,17,20,37]. Although spatial and temporal dependence of cellular proliferation was investigated [16,19,20], systematic and detailed dynamic studies on cellular proliferation and glial scar formation were lacking. We designed a ‘time-block’ Brdu injection protocol to precisely label the proliferating cells at different times after PT. Our results show that Brdu+ proliferating cells and GFAP+ Brdu+ proliferating reactive astrocytes were generated at the highest rate within post ischemic days 3 to 4 and then quickly reduced, and that proliferating astrocytes only accounted for small percentage of Brdu+ cells in each time period studied with the highest percentage being 5.8%. Thus our results demonstrated that the majority of GFAP+ reactive astrocytes resulted from the upregulation of GFAP in existing astrocytes without proliferation, and that the generation of proliferating astrocytes was highly time-dependent during post ischemia. The low proliferating rate of reactive astrocytes after a stroke is consistent with a report from Barret et al. although they used a different Brdu labeling protocol and ischemic model [20], in addition, despite the agreement of their results with ours, they did not conduct a detailed time course study.

On the other hand, the results of our study also revealed the persistence of a high GFAP+ cell density even in the distant region from ischemic core up to two weeks after PT, although the rate of GFAP+ Brdu+ cell generation was dramatically reduced after day 6. Glial scar is clearly formed from day 6 after ischemia. The GFAP+ reactive astrocytes experienced significant changes in morphology characterized as hypertrophy and stellate before day 6, as they became densely packed and formed a stream with straight and elongated processes pointing towards the ischemic core after day 6, thereby sharply separating the ischemic core area from unaffected issue.

Besides astrocytes, microglia also played a crucial role in cellular proliferation and glial scar formation post stroke. As a major source of proinflammatory factors in brain, microglia is significantly activated after ischemic stroke [18,19]. Our results showed that the dynamic changes of Iba1+ cells and Iba1+ Brdu+ cells followed the same pattern as GFAP+ and GFAP+ Brdu+ cells, i.e., with the highest rates recorded within post ischemic days 3 to 4. Nevertheless, a large increase in Iba1+ Brdu+ microglia also took place within post ischemic days 1 to 2, and the response receded more quickly compared with GFAP+ reactive astrocytes with undetectable proliferating Iba1+ microglia from day 10 after ischemia. Thus, compared with astrocytes, Iba1+ microglia recruits to ischemic regions more rapidly in response to ischemia. Another difference between astrocytes and microglia is that the percentage of proliferating microglia from total proliferating cells is much higher than the percentage of proliferating reactive astrocytes after PT. This is consistent with a recent study showing that the resident microglia, not infiltrated macrophages, contribute to proliferating microglia after PT [28]. Microglia has been described to play both beneficial and detrimental roles in stroke [18]. The rapid recruitment of microglia is consistent with the observation that microglia are the first barrier of the central nervous system, respond immediately to exogenous stimulation through increased inflammatory signals thereby contributing to the destruction of the infectious agents to protect sensitive neural tissue by removing cell debris and engulfing invading neutrophil granuocytes [38,39]. Together with the same time course of lesion expansion and edema and swelling, our data suggest that the first few days after ischemia is a critical time window for microglia to exert beneficial or detrimental effects after ischemia. In addition, the percentage of proliferating Iba1+ microglia in total Brdu+ cells was much larger than that of proliferating GFAP+ cells. Since Iba1 is also expressed in macrophages [20,40], the infiltration of macrophage might contribute greatly to total Iba1+ cells after ischemia, but on the other hand, this also suggests that stroke-induced increase of proliferated microglia might play a role in maintaining their continuous surveillance function and supporting glial scar formation. After day 6, densely packed Iba1+ cells in the ischemic core region were observed concomitant with astrocyte-derived scar formation, suggesting that both astrocytes and microglia contribute to scar formation.

Based on our Brdu+ labeling protocol, no mature neurons (Brdu+/NeuN+) were found in the penumbra. This is probably because the Brdu labeling time (i.e., two days between Brdu+ injection and transcardial perfusion in our protocol) is too short to form mature neurons in the penumbra in the cortex. However, mature neurons can be found in the cortex under normal and ischemic conditions using protocols with multiple daily injections of Brdu and prolonged labeling and waiting periods before immunocytochemical study [41-43]. On the other hand, although we did not examine the time course of apoptotic neuronal death, it is time dependent from our and other study based on TUNEL and caspase-3 staining [12,20].

After ischemia, the brain will experience spontaneous recovery process [3,31,44]. However, it is still poorly understood how infarct expansion, cellular proliferation and reactive gliosis are correlated with functional deficits. Our data from behavioral study showed that PT significantly affected forelimb shift asymmetricity, strength, and sensory motor impairments in post ischemic days 2–4, which is in a similar time window with the infarct expansion, brain edema and swelling formation, and the highest rates of cell proliferation and reactive astrocyte generation. It is still controversial whether glial scar has beneficial or detrimental effects on neuronal and brain protection after ischemia. Attenuation of glial scar formation has been associated with both increases and decreases in infarct volumes and functional recovery [12,15,21,37]. Double knockout mice of GFAP and vimentin have larger infarction than WT mice after ischemia [45]. The current study shows that functional deficit is recovered from day 6 after ischemia when glial scar starts to form, suggesting that glial scarring might have a beneficial effect by stopping the expansion of the ischemic core. Although there have been reports regarding behavioral recovery after photothrombotic ischemia in mice, it appears that these behavioral tests have only been conducted after day 7 of post ischemia or were only conducted for a single post ischemic time point in past studies [3,4,46], and consequently missed critical information in the acute phase and during recovery process. Our study revealed that brain damage including infarct expansion and brain swelling as well as edema, and cellular proliferation are positively correlated with behavioral deficits in the acute phase. Behavioral recovery starts 6 days after PT when infarct expansion, brain edema and swelling, and cellular proliferation are decreased.

## Conclusions

In clinic, the therapeutic aim is to salvage the cells in the penumbra; thus, understanding molecular and cellular events is critical for providing insights for therapeutic strategy. The present paper provides a systematic study on the time courses of brain injury, cellular responses and behavioral deficits after PT-induced brain ischemia in adult mice. The astrocytes and microglia around the lesion respond to ischemic insults with a spatiotemporal dependent proliferation, which highly correlates with brain lesion progress and swelling and behavioral deficits. Our results suggest that dynamic cellular proliferation and reactive astrogliosis might determine the progress of brain and neuronal remodeling and functional recovery, and that targeting reactive astrocytes is an important strategy to facilitate improvement of stroke outcomes.

## Competing interests

The authors declare no competing interests.

## Authors’ contributions

SD conceived of the study, and supervised and participated in designing the experiments, and wrote the manuscript. HL generated ischemic mice and analyzed infarct volumes and performed behavioral tests. He also helped to draft manuscript. NZ performed immunostaining and analyzed the related data. HYL and YY participated in behavioral tests. QC and LM performed MRI experiments. All authors read and approved the final manuscript.

## Supplementary Material

Additional file 1: Figure S1 Neuronal density in the penumbra after PT. **Figure S2.** Brdu labeling cells in the contralateral hemisphere of ischemic mice. Click here for file
