# Supplementary material for: Histological, cellular and behavioral assessments of stroke outcomes after photothrombosis-induced ischemia in adult mice
Source: BMC Neurosci. 2014 May 2;15:58. doi: 10.1186/1471-2202-15-58 (PMC4039545; doi:10.1186/1471-2202-15-58)
Supplement: Additional file 1: Figure S1 — Neuronal density in the penumbra after PT. Figure S2. Brdu labeling cells in the contralateral hemisphere of ischemic mice. [file 1471-2202-15-58-S1.doc]

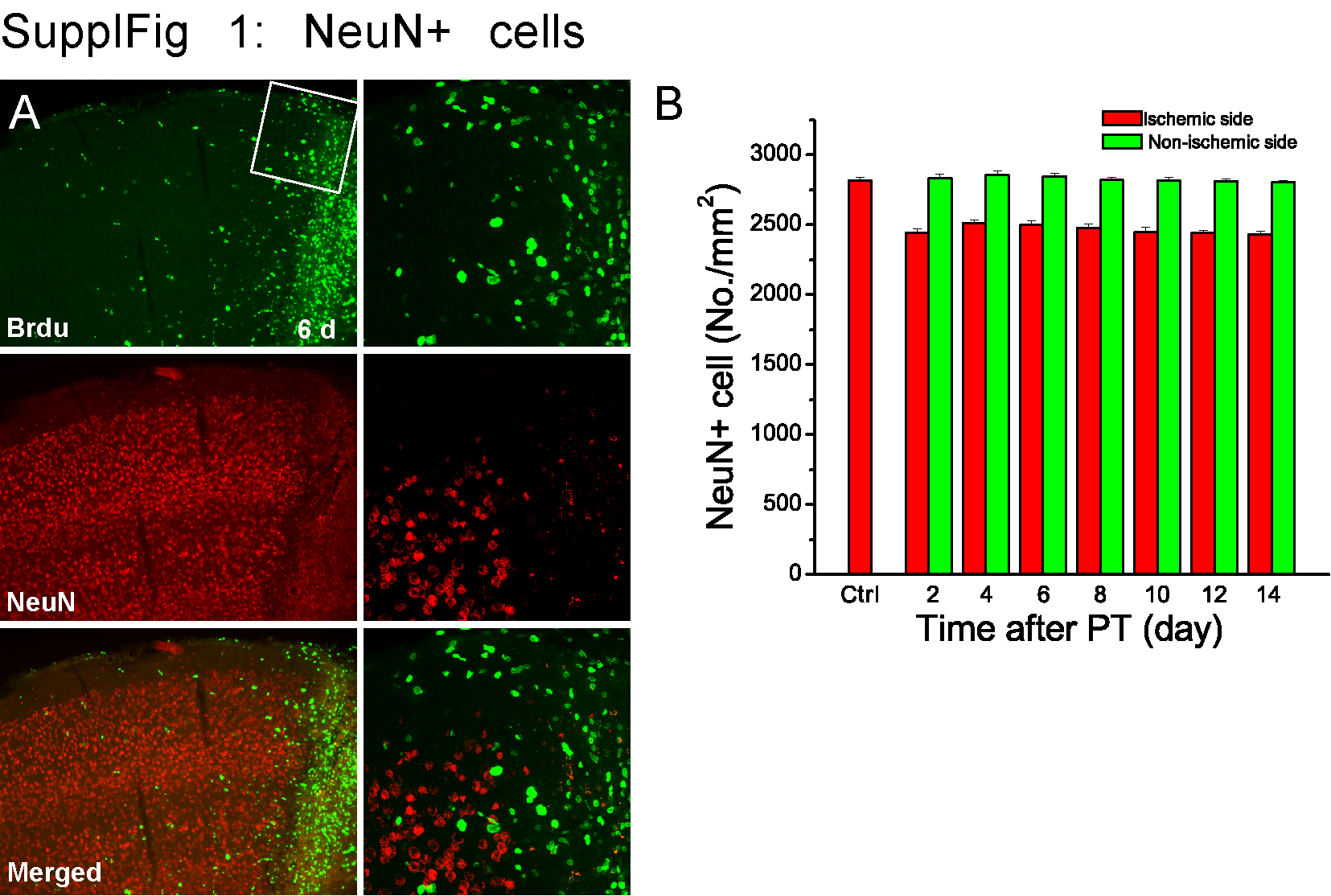
 **Additional file 1: Figure S1.** **Neuronal density in the penumbra after PT.** A) Representative fluorescent images of NeuN and Brdu double stained brain sections from mice at day 6 after PT. B) The density of NeuN+ cells in the penumbra. The cells were counted in the penumbral region of 200 μm×400 μm in layers 2/3 cortex from the edge of ischemic core.


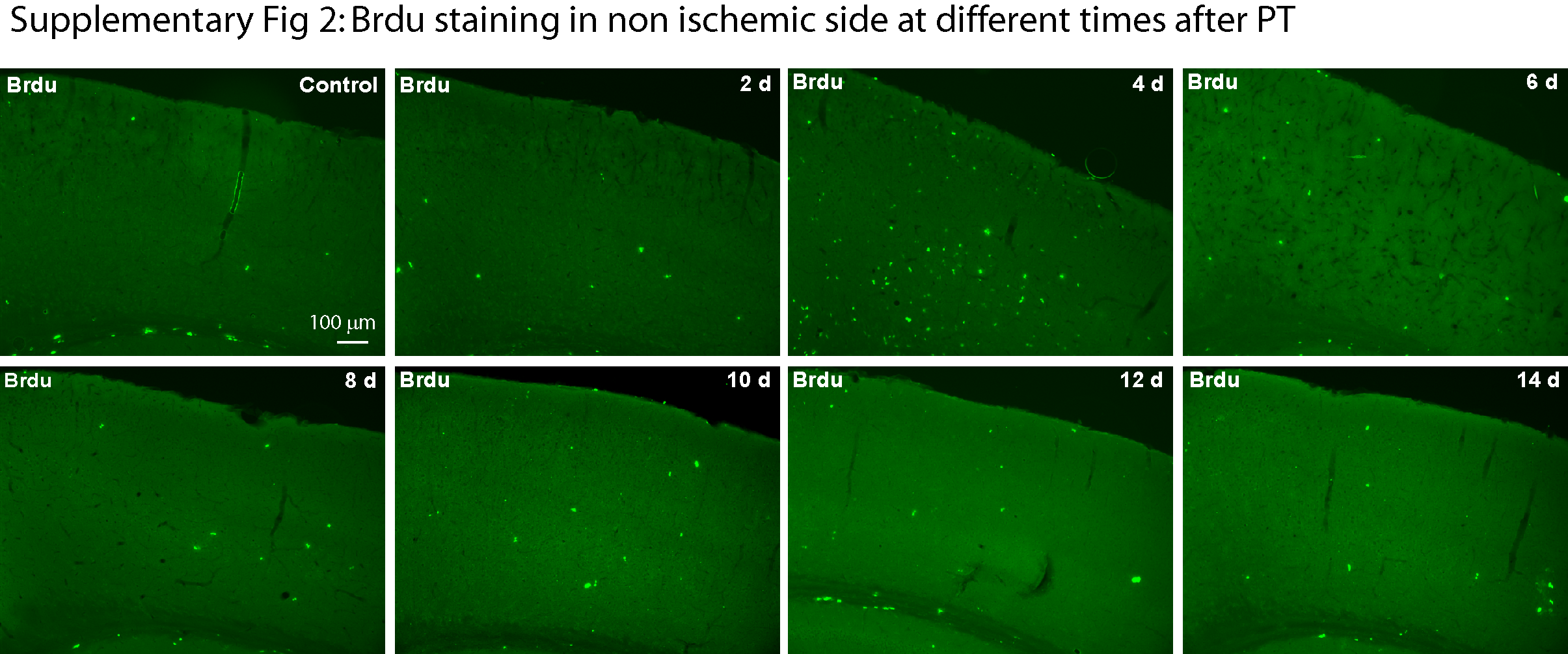


**Additional file 1: Figure S2. Brdu labeling cells in the contralateral hemisphere of ischemic mice.** Images of Brdu staining in the cortices of control mouse and the contralateral hemispheres of ischemic mice at different times after PT. Notice the high density of Brdu+ cells from mice four days after PT.
